# A snapshot of women’s and clinicians’ perceptions of the double balloon catheter for induction of labor

**DOI:** 10.18332/ejm/146689

**Published:** 2022-05-30

**Authors:** Sarah Waldron, Hannah Contziu, Olga Aleshin, Hala Phipps

**Affiliations:** 1 RPA Women and Babies, Royal Prince Alfred Hospital, Sydney, Australia; 2 Sydney Institute for Women, Children and their Families, Sydney Local Health District, Sydney, Australia; 3 School of Nursing and Midwifery, Faculty of Health, University of Technology Sydney, Sydney, Australia; 4 Susan Wakil School of Nursing and Midwifery, Faculty of Medicine and Health, University of Sydney, Sydney, Australia

**Keywords:** labor and birth, cervical ripening, pregnant women, induction of labor, double-balloon catheter

## Abstract

**INTRODUCTION:**

Induction of labor (IOL) is rising globally and is growing steadily in the state of New South Wales, Australia. There are numerous methods of induction of labor, including the double balloon catheter (DBC). There is minimal evidence on women’s attitudes and experiences and clinician’s opinions on the use of the DBC. This study aims to explore the views regarding DBC insertion and effectiveness from women induced with a DBC and clinicians involved in the catheter insertion and care.

**METHODS:**

This study is a descriptive survey of two prospective, de-identified, self-reported questionnaires which were completed in 2016. One questionnaire was administered to term pregnant women that were admitted to the antenatal ward post IOL, and the other was completed by midwives and obstetric doctors working in the ward at the time.

**RESULTS:**

The DBC appeared to be a well-accepted method of cervical ripening among women (61%) and clinicians (>82%). Success of DBC to achieve an artificial rupture of membrane post removal, directly correlates to women’s acceptance (61%). While most clinicians (59–67%) perceived insertion of DBC in an outpatient setting and then women discharged home was appropriate, only 13% of women were in favor. On the logistics of the procedure in respect to insertion and removal of the DBC, there were differences of opinion, with only 43% of women, 77% of midwives and 33% of doctors stating that the timing of insertion and removal needed to be improved.

**CONCLUSIONS:**

This study highlights the need to undertake qualitative research to further explore women’s views and perceptions on IOL in order to ensure that clinical practice is woman-centered and evidenced-based, and to guide policy and protocol.

## INTRODUCTION

Induction of labor is rising globally and is growing steadily in the state of New South Wales, Australia. In 2011, 26.5% of labors were induced compared to 31.1% in 2016. The most common reasons for induction of labor were prolonged pregnancy, gestational diabetes and prelabor rupture of membranes^1^.

There are numerous methods of induction of labor such as membrane sweeping, amniotomy, prostaglandins, balloon catheters and the double balloon catheter (DBC). Balloon catheters for cervical ripening are well established in the literature as being both effective and safe^2-5^. While balloon catheters often take longer to ripen the cervix, they have a better safety profile: reduced hyperstimulation, tonic contractions and fetal heart rate changes^2,6-10^.

There is minimal evidence on women’s attitudes and experiences of double balloon catheter use and clinician’s opinions of catheter effectiveness and ease of use. Numerous studies have explored the experiences of women who have had an induction of labour^11-14^. In contrast, there is minimal research which has investigated women’s and clinician’s views of the double balloon catheter as an induction of labor process^15^.

Interestingly, motivation for this study was the result of anecdotal reports from midwives about workload issues, namely the extended waiting periods of women being induced and the timing and delays of double balloon catheter insertion and removal. Midwives at the hospital are not accredited in the insertion of a double balloon catheter and often need to wait for an available doctor to perform the procedure.

The aim of this study was to explore the views regarding double balloon catheter insertion and effectiveness from women being induced with the catheter and the clinicians involved in the catheter insertion and care.

## METHODS

This is a prospective descriptive survey of two de-identified, self-reported questionnaires, that was undertaken between September and November 2016, in a tertiary hospital in Sydney, NSW, Australia. Two de-identified, self-reported questionnaires were distributed (Supplementary file). One was administered to women (n=26) of term gestation with a singleton pregnancy that were admitted to the antenatal ward for an induction of labor (at post double balloon catheter removal) and the other was administered to midwives or doctors (n=42) involved in the double balloon catheter insertion and care of the women.

**Figure 1:**
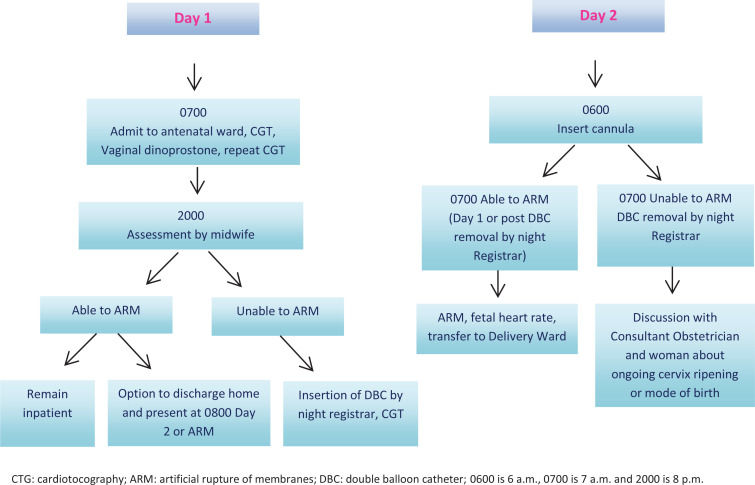
Induction of labor process at a tertiary hospital, Sydney, Australia

The double balloon catheter is a soft, thin tube that is inserted vaginally through the cervix, two balloons are inflated, one on the side of the internal os (orifice) providing pressure and overstretching the lower uterine segment and indirectly causing localized prostaglandin^6^. The other balloon is inflated on the side of the external os, pressure is applied and dilation of cervix results from this pressure on both sides^6^. Cervical ripening was performed with 0.3 mg/h slow release intravaginal dinoprostone over 12 hours for a Bishop’s score <7. If the Bishop score was still <6, a Cook® cervical ripening balloon was routinely inserted into the cervix, the balloons filled as per the manufacturer’s instructions and removed after another 12 hours. Induction of labor was by artificial rupture of the membranes prior to commencing an oxytocin infusion. The key indications for induction of the study setting included gestational diabetes, reduced fetal movements, prolonged pregnancy and prelabor rupture of membranes^16^. Figure 1 provides an overview, step by step, of what occurs when a woman is admitted to the antenatal ward for induction of labor.

### Data collection and analysis

We collected data relating to the views of women and clinicians regarding double balloon catheter insertion and effectiveness. The catheter was inserted either digitally or via visualization of the cervix using a speculum at the clinician’s discretion. We did not collect data on individual practitioner insertion styles. Double balloon catheter placement time varied however; most occurred between 9 p.m. and 1 a.m. The variation was due to the heavy workload of the medical staff which delayed both medical review and insertion of vaginal prostaglandin in the morning, and midwives being not accredited to perform catheter insertion. Clinical data were collected on women’s views on method and effectiveness of induction of labor, pain relief, artificial rupture of membranes, opinions on having the double balloon catheter in a future pregnancy as well as the option of the insertion as an outpatient. The effectiveness of the double balloon catheter was determined to be the successful artificial rupture of membranes after removal of the double balloon catheter. Clinical data were also collected from clinicians directly involved in both the insertion and care of the double balloon catheter. These data included the views of clinicians on ease of insertion, effectiveness, insertion and removal timing, and the option of the double balloon catheter as an outpatient measure. Demographic data were collected from the maternity database.

Survey data were analyzed using frequency tabulations. Oral and written information on the study was provided to all participants, who then provided voluntary oral consent to participate. The study had local ethical clearance from the Sydney Local Health District Ethics Review Committee (RPAH Zone).

## RESULTS

The mean age of the women participants was 31 years, pre-pregnancy body mass index was 23 (kg/m^2^), and the majority (≥58%) of women were born in a country other than Australia and New Zealand^16^. Women with diabetes in pregnancy and women born in South Asia were more likely to be induced than other women. Women born in South-East Asia were more likely to go into spontaneous labor at 38^(+0/7)^ to 39^(+6/7)^ weeks’ gestation and women born in Australia or New Zealand were more likely to progress beyond 40 completed weeks’ gestation^16^. Of the 42 clinicians who participated in the survey, 64% (n=27) were midwives, 29% doctors (n=12) and 7% student midwives (n=3) (Table 1). Most midwives, doctors and midwifery students were familiar with the hospital’s induction of labor policy, 93% (n=25), 92% (n=11), and 100% (n=3), respectively (Table 2).

**Table 1:** Breakdown of clinician study participants’ experience in a tertiary hospital, Sydney, Australia (N=42)

*Experience*	*n (%)*
**Midwifery years of experience** (n=30)	
<5	13 (43)
5–10	9 (30)
>10	8 (27)
**Medical staff experience** (n=12)	
RMO	1 (8)
SRMO	2 (17)
Registrar	7 (58)
Senior Registrar	1 (8)
Senior staff specialist	1 (8)

RMO: resident medical officer. SRMO: stream resident medical officer (obstetrics stream). RMO and SRMO are medical doctors 2–3 years postgraduate. Registrar and Senior Registrar are 4–9 years postgraduate in a specialty training program (i.e. obstetrics). Staff Specialist is a consultant obstetrician and gynecologist.

**Table 2:** Clinicians’ responses to the survey, in a tertiary hospital, Sydney, Australia (N=42)

*Responses*	*Midwives (n=27) n (%)*	*Doctors (n=12) n (%)*	*Student midwives (n=3) n (%)*
Familiarity with RPAH IOL policy	25 (93)	11 (92)	3 (100)
Believe the DBC is easy to insert	8 (4)	8 (67)	0
Believe the DBC is effective at ripening the cervix on most occasions	22 (82)	10 (83)	1 (33)
Feel timing of insertion and removal of DBC needs to be improved	21 (77)	4 (33)	3 (100)
Believe the DBC is an appropriate outpatient measure	16 (59)	8 (67)	2 (67)

RPAH: Royal Prince Alfred Hospital. IOL: induction of labor. DBC: double balloon catheter.

The majority (96%, n=22) of women having an induction of labor, had prostaglandin and 61% (n=14) had an artificial rupture of membranes performed successfully. On the issue of pain, 100% (n=23) reported that the insertion of the catheter was painful (Table 3). Most of the women (87%, n=20) required pain relief post double balloon catheter insertion and 75% (n=15) stated that the pain relief was effective (Table 3).

**Table 3:** The responses of the pregnant women study participants that were induced and had a double balloon catheter inserted, in a tertiary hospital, Sydney, Australia (N=23)

*Responses*	*n (%)*
Had vaginal prostaglandin in IOL	22 (96)
Was insertion of DBC painful	23 (100)
Pain relief required post DBC insertion	20 (87)
Pain relief effective	15/20 (75)
ARM performed successfully post DBC removal	14 (61)
DBC inserted and removed at an appropriate time of day	10 (43)
Discharge home with DBC in situ as an outpatient	3 (13)
Consent to DBC in future if require IOL	14 (61)

IOL: induction of labor. DBC: double balloon catheter. ARM: artificial rupture of membranes.

Doctors and midwives consistently believed that the double balloon catheter to be effective in ripening the cervix on most occasions, 83% (n=10) and 81.5% (n=22) respectively, compared to 33% of student midwives (n=1) (Table 2). Sixty-one percent (n=14) of women said they would consent to double balloon catheter insertion in the future and this result was consistent with the 61% (n=14) who reported that an artificial rupture of membranes could be performed post catheter removal (Table 3).

The double balloon catheter was perceived as appropriate in an outpatient setting by most clinicians, 59% of midwives (n=16), 67% (n=8) of doctors, and 67% (n=2) of student midwives (Table 2). When the women were asked if they would be happy to be discharged home with double balloon catheter *in situ*, only 13% (n=3) agreed (Table 3).

On the issue of the timing of double balloon catheter insertion and removal, 77% (n=21) of midwives felt timing of insertion and removal needed to be improved compared to 33% (n=4) of doctors (Table 2). Only 43% of women (n=10) reported insertion and removal was performed at an appropriate time of day (Table 3).

## DISCUSSION

The double balloon catheter for induction of labor was well-accepted by the majority of the women and clinicians surveyed at a tertiary hospital in the state of New South Wales, Australia. Our study found that the catheter was a well-accepted method with the majority of the pregnant women reporting that they would consent to the procedure in a future pregnancy, if clinically indicated. This finding is consistent with the results of another study^15^ that found that women receiving the double balloon catheter, in combination with oral misoprostol, would recommend the method to others.

The majority of midwives and doctors agreed that on most occasions the double balloon catheter was effective as a cervical ripening device. This finding is consistent with current literature that the double catheter is more effective than prostaglandins^2,8,10,17^.

Our study highlights an inconsistency between clinician’s perceptions about timing of double balloon catheter insertion and removal. The majority of midwives agreed that this needed to be improved, while only a third of doctors felt the same way. Interestingly, motivation for this study was the result of anecdotal reports from midwives about workload issues, namely the extended waiting periods of women being induced and the timing and delays of double balloon catheter insertion and removal. Possible solutions to these delays include: a designated treatment room, scheduling systems for catheter insertions and organized staff training, especially for upgrading the skills of midwives^4^.

This study found that women consistently regarded the double balloon catheter insertion as painful to varying degrees, with some describing the pain as unbearable. The pain experienced compared to other cervical ripening methods varies between studies. Wilkinson et al.^4^ reported that the double balloon catheter is often more painful than other cervical ripening methods. Interestingly, in the study of Jozwiak et al.^10^, women reported the highest levels of discomfort with prostaglandins, then the double balloon catheter, and the lowest discomfort with the single balloon catheter. Similarly, in the study of Pennell et al.^9^, women reported higher levels of pain with both the double balloon catheter and prostaglandins compared to the single balloon catheter. Although our study did not compare the catheter to other methods of induction of labor, our findings are consistent with the current literature.

The majority of women in our study reported that the pain relief offered to them post insertion was effective. This finding is similar to that of Kehl et al.^15^, where women were not bothered by the double balloon catheter once *in situ*. Insertion using digital technique is reported as slightly more acceptable compared to insertion with a speculum^4^. Similarly, digital insertion technique is associated with significantly higher tolerance of single balloon catheter insertion^18^. This is an area for further research.

When it comes to outpatient setting for cervical ripening with double balloon catheter, our findings are inconsistent with the literature, for the pregnant women but not the clinicians. In regard to the perceived acceptability, the majority of clinicians considered this to be appropriate whereas women did not. Double balloon catheter in the outpatient setting has been associated with improved relaxation, privacy and sleep, compared to women in the inpatient setting^4^. Most importantly, double balloon catheter in the outpatient setting has been found to be as safe and effective as standard inpatient care^4,19-22^. Furthermore, the outpatient setting allows women the benefit of the comfort of their own home without increasing their anxiety^23^.

The evidence for outpatient double balloon catheter insertion indicates better acceptability by pregnant women, contrary to our findings. We propose several reasons for the contrasting views between clinicians and women. Firstly, double balloon catheter management in the outpatient setting may not have been clearly explained or discussed with the women we surveyed. Women may have been unaware that double balloon catheter in the outpatient setting depended on being identified as ‘low-risk’, with normal fetal heart rate patterns prior to discharge, follow-up at the hospital the next day, and clear guidance on when to call the hospital, i.e. rupture of membranes. Secondly, some women may have cultural perceptions that the hospital is safer than home. Finally, some of the women surveyed may have been induced for reasons deemed high-risk, as our selection criteria did not differentiate between high-risk and low-risk induction of labor.

### Limitations

The study had some limitations. Firstly, the sample size was small which makes it difficult to draw any major conclusions. We did not differentiate between primiparous and multiparous women. This distinction may have highlighted a difference in views to note for future induction of labor planning. Lastly, it was difficult to track exactly how many women were administered a double balloon catheter in the study period due to a lack of clear documentation in the maternity database.

## CONCLUSIONS

Our study found the double balloon catheter to be a well-accepted method of cervical ripening among women and clinicians, while revealing differing attitudes between women and clinicians regarding timing and outpatient cervical ripening. Considering the increasing rates of induction of labor, further research into the acceptability of double balloon catheter for cervical ripening is required. Our study contributes to the current literature but highlights the need for further investigation into outpatient options. Additionally, it highlights the need to undertake qualitative research to further explore women’s views, experiences and perceptions on induction of labor, to ensure clinical practice is woman-centered and evidenced-based, and to guide policy and protocol.

## Data Availability

The data supporting this research are available from the authors on reasonable request.
